# Charting the Progress of Epilepsy Classification: Navigating a Shifting Landscape

**DOI:** 10.7759/cureus.46470

**Published:** 2023-10-04

**Authors:** Alaa Abdelsamad, Meet Popatbhai Kachhadia, Talha Hassan, Lakshya Kumar, Faisal Khan, Indrani Kar, Uttam Panta, Wirda Zafar, FNU Sapna, Giustino Varrassi, Mahima Khatri, Satesh Kumar

**Affiliations:** 1 Research and Development, Michigan State University, East Lansing, USA; 2 Internal Medicine, PDU (Pandit Dindayal Upadhyay) College, Civil Hospital Campus, Rajkot, IND; 3 Internal Medicine, KEMU (King Edward Medical University) Mayo Hospital, Lahore, PAK; 4 General Medicine, PDU (Pandit Dindayal Upadhyay) Medical College, Rajkot, IND; 5 Medicine, Dow University of Health Sciences (DUHS), Karachi, PAK; 6 Medicine, Lady Hardinge Medical College, New Delhi, IND; 7 Medicine, Chitwan Medical College, Bharatpur, NPL; 8 Medicine, University of Medicine and Health Sciences, Toronto, CAN; 9 Pathology, Albert Einstein College of Medicine, New York, USA; 10 Pain Medicine, Paolo Procacci Foundation, Rome, ITA; 11 Medicine and Surgery, Dow University of Health Sciences (DUHS), Karachi, PAK; 12 Medicine and Surgery, Shaheed Mohtarma Benazir Bhutto Medical College, Karachi, PAK

**Keywords:** artificial intelligence in epilepsy, precision medicine, genetic insights, neuroimaging advances, seizure phenomenology, epilepsy classification

## Abstract

Epilepsy, a neurological disorder characterized by recurrent seizures, has witnessed a remarkable transformation in its classification paradigm, driven by advances in clinical understanding, neuroimaging, and molecular genetics. This narrative review navigates the dynamic landscape of epilepsy classification, offering insights into recent developments, challenges, and the promising horizon. Historically, epilepsy classification relied heavily on clinical observations, categorizing seizures based on their phenomenology and presumed etiology. However, the field has profoundly shifted from a symptom-based approach to a more refined, multidimensional system. One pivotal aspect of this evolution is the integration of neuroimaging techniques, particularly magnetic resonance imaging (MRI) and functional imaging modalities. These tools have unveiled the intricate neural networks implicated in epilepsy, facilitating the identification of distinct brain abnormalities and the categorization of epilepsy subtypes based on structural and functional findings. Furthermore, the role of genetics has become increasingly prominent in epilepsy classification. Genetic discoveries have not only unraveled the molecular underpinnings of various epileptic syndromes but have also provided valuable diagnostic and prognostic insights. This narrative review delves into the expanding realm of genetic testing and its impact on tailoring treatment strategies to individual patients. As the classification landscape evolves, there are accompanying challenges. The narrative review underscores the transformative potential of artificial intelligence and machine learning in epilepsy classification. These technologies hold promise in automating the analysis of complex neuroimaging and genetic data, offering enhanced accuracy and efficiency in epilepsy diagnosis and classification. In conclusion, navigating the shifting landscape of epilepsy classification is a journey marked by progress, complexity, and the prospect of improved patient care. We are charting a course toward more precise diagnoses and tailored treatments by embracing advanced neuroimaging, genetics, and innovative technologies. As the field continues to evolve, collaborative efforts and a holistic understanding of epilepsy's diverse manifestations will be instrumental in harnessing the full potential of this dynamic landscape.

## Introduction and background

Epilepsy is a neurological ailment that is defined by the occurrence of recurrent unprovoked seizures. It is one of the first recognized medical conditions in human history, with allusions to its existence extending back to ancient civilizations. The subject's longevity has made it a topic of great fascination, enigma, and scientific investigation [1]. Extensive study has been conducted on epilepsy due to its numerous symptoms, complex origin, and profound influence on the lives of the persons affected. As a result, substantial breakthroughs have been made in our understanding of this ailment. The historical narrative surrounding epilepsy has been characterized by several fallacies and the perpetuation of stigmatization, primarily stemming from an absence of understanding of its fundamental mechanics. Hippocrates, a prominent figure in medicine during the fifth century BCE, significantly contributed to the comprehension of epilepsy. He proposed that this condition could have origins in the brain, challenging the prevailing belief that supernatural influences caused it. Nevertheless, it was not until the contemporary age that epilepsy was perceived as a neurological condition characterized by discernible physiological foundations [2]. The alteration in perspective has been crucial in advancing epilepsy categorization and its essential function in diagnosing and treating the condition. The classification of epilepsy has a fundamental role in establishing a solid foundation for the implementation of successful medical interventions and the advancement of research endeavors. The precise classification of epileptic syndromes and seizure types not only enhances the exchange of information among healthcare practitioners but also substantially impacts treatment choices. This study offers crucial information regarding the prognosis, treatment response, and probable co-occurring medical conditions [3]. The classification of epilepsy serves as a crucial tool for doctors, guiding their decision-making processes. Additionally, it serves as a roadmap for researchers, shaping their activities in the field. Moreover, it represents a beacon of hope for persons impacted by epilepsy, illuminating the route toward improved understanding and management of the condition. The main objective of this extensive literature review is to examine the difficult field of epilepsy categorization, providing a thorough analysis of its historical development, current frameworks, and the various elements that contribute to its complexity. In this complex and diverse context, we shall explore various overarching themes that encompass crucial aspects of epilepsy classification. The historical progression of epilepsy classification is evidence of the persistent endeavor to comprehend and categorize this mysterious condition [4]. Epilepsy has been ascribed to divine retribution, demonic influence, and even paranormal capabilities. The proposition that epilepsy may have its origins in the brain was initially put out by Hippocrates, thereby marking a significant advancement toward a more scientific perspective. Nevertheless, the progression of medical knowledge beyond superstitious beliefs and folklore was a process that spanned several centuries. The Enlightenment period was a significant juncture at which notable figures such as Jean-Marc Gaspard Itard and John Hughlings Jackson commenced elucidating the neurological underpinnings of epilepsy. This section aims to provide a complete account of the historical development of epilepsy classification, emphasizing significant milestones and influential figures that have contributed to our current understanding. Over the past few decades, there has been a notable shift in the categorization of epilepsy, transitioning from a method that mostly relies on symptomatology to a more comprehensive and multifaceted framework. The International League Against Epilepsy (ILAE) has been instrumental in establishing current epilepsy classification systems. The ILAE classification was a notable shift from prior methodologies, emphasizing the significance of seizure semiology, etiology, and syndrome classification [5]. This section will explore the fundamental ideas and criteria that form the basis of contemporary epileptic classification systems. It aims to elucidate the role of these systems in facilitating enhanced precision in the diagnosis and treatment of epilepsy. Examining and depicting different types of seizures and their corresponding characteristics, known as seizure phenomenology, plays a crucial part in categorizing epilepsy. A comprehensive comprehension of the subtle distinctions among various types of seizures and their observable symptoms is required to achieve precise classification and prognosis [6]. This section examines the importance of seizure phenomenology in classifying epilepsy, emphasizing the role of comprehensive seizure observations in distinguishing between different epilepsy syndromes. Neuroimaging has experienced significant progress, transforming our capacity to investigate the anatomical and functional characteristics of the brain in persons diagnosed with epilepsy. Advanced imaging techniques, including magnetic resonance imaging (MRI), positron emission tomography (PET), and functional MRI (fMRI), have yielded unparalleled knowledge regarding the neurological underpinnings of epilepsy [4]. This paper explores the importance of neuroimaging in classifying epilepsy, focusing on using these methods in identifying specific brain abnormalities and categorizing different subtypes of epilepsy. The field of genetics has assumed a significant role in the categorization of epilepsy. In recent years, there have been significant advancements in identifying particular gene abnormalities linked to different epilepsy syndromes [7]. The comprehension of the genetic foundations of epilepsy facilitates the improvement of diagnostic precision and has the potential for the development of tailored treatment strategies. This section aims to examine the growing domain of genetic testing and its implications for the classification and therapy of epilepsy. The notion of precision medicine, which involves customizing treatment approaches based on the unique characteristics of individual patients, has garnered significant traction within the realm of epilepsy. The field of precision medicine acknowledges the inadequacy of a uniform approach in managing epilepsy and emphasizes the significance of precise categorization in informing treatment choices [3]. This study explores the impact of enhanced classification techniques on the advancement of tailored treatment strategies, maximizing therapeutic efficacy for patients diagnosed with epilepsy. Artificial intelligence (AI) and machine learning (ML) have become prominent techniques in classifying epilepsy in the era of technology and data-driven healthcare [6]. Utilizing these technologies presents the opportunity to automate the examination of intricate neuroimaging and genetic data, augmenting the precision and effectiveness of diagnosing and categorizing epilepsy. This section examines the incorporation of AI in classifying epilepsy and its potential ramifications for the future of epilepsy management. As we commence our investigation into the categorization of epilepsy, we acknowledge the significant influence that precise classification exerts on the well-being of those affected by epilepsy. Engaging in this pursuit is not solely an academic undertaking but rather a crucial component of providing compassionate and efficient healthcare. Through an exploration of the historical progression, current frameworks, and emerging technologies in the domain of epilepsy classification, this review aims to enhance comprehension of this complex disorder and stimulate continuous endeavors to enhance the well-being of individuals impacted by epilepsy.

## Review

Methods

This narrative review largely relies on a comprehensive literature search and synthesis of pertinent research articles, clinical guidelines, and historical documents to examine the strategies utilized for tracking the advancement of epilepsy classification and adapting to the changing landscape. The evaluation contains a wide range of materials, including peer-reviewed journal articles, books, and reports, emphasizing influential publications and recent developments in epilepsy classification. The first step in our research consisted of performing systematic searches in significant databases, including PubMed, Scopus, and Web of Science. A combination of pertinent keywords and controlled vocabulary terms were utilized, encompassing subjects such as "epilepsy classification," "seizure phenomenology," "neuroimaging advances," "genetic insights," "precision medicine," and "artificial intelligence in epilepsy." The utilization of Boolean operators and truncation was done to achieve a comprehensive retrieval of relevant material. We implemented specific criteria for inclusion and removal to uphold the credibility and quality of the sources used in this narrative review. The inclusion criteria of this study comprised a range of sources, including studies, guidelines, and historical documents. These sources were selected based on their significant contributions to understanding epilepsy categorization. Such contributions could be significant to historical context, pivotal insights into classification systems, or contemporary improvements. The application of exclusion criteria was employed to filter out studies that did not pertain to the core aims of this review, specifically those that focused on elements of epilepsy unrelated to classification. The synthesis of the literature involved a thorough analysis of the identified articles and documents, focusing on the historical development of epilepsy classification, current classification systems such as the ILAE classification, the significance of seizure phenomenology, advancements in neuroimaging, genetic discoveries, the application of precision medicine, and the incorporation of AI in the classification of epilepsy. The process involved the extraction, organization, and synthesis of data to construct a cohesive narrative that effectively encompasses the core aspects of each theme and their significance within the broader framework of epilepsy classification. In addition to conducting a comprehensive literature search, this narrative review incorporates the writers' expertise and experience in epilepsy and neurology. The writers have significantly contributed to epilepsy categorization and associated areas, including clinical practice, research, and academic discourse. Their extensive expertise and critical analysis have greatly enhanced the review process. It is crucial to acknowledge that this review incorporates diverse sources and approaches. However, it is essential to recognize that it follows the narrative review structure, prioritizing the amalgamation and interpretation of existing information rather than a systematic and quantitative study of data. Using the narrative technique facilitates a thorough examination of the topic, providing valuable perspectives on the historical backdrop, current advancements, and potential future directions of epilepsy classification in a logical and enlightening fashion.

Historical evolution of epilepsy classification

Epilepsy, a condition acknowledged since ancient times, has had a significant evolution in its categorization throughout the decades. The classification of epilepsy has undergone significant historical development, mirroring the increased comprehension of this intricate neurological condition [8]. The subsequent part will examine the historical methodologies employed in classifying epilepsy, emphasizing significant milestones and modifications in classification systems throughout history.

The Study of Ancient Beliefs and Their Reliance on Supernatural Explanations

The historical narrative of epilepsy is closely connected with a complex tapestry of superstitions, myths, and supernatural beliefs. Epilepsy was commonly perceived as a mystical or divine occurrence in ancient civilizations, including Mesopotamia, Egypt, and Greece. Epileptic seizures were commonly ascribed to the influence of deities, evil entities, or supernatural forces. The ancient Greeks, specifically, attributed epilepsy to supernatural possession, therefore coining the name "the sacred disease." Hippocrates, widely recognized as the progenitor of Western medicine, questioned the validity of these supernatural convictions and established the fundamental principles for a more empirical comprehension of epilepsy [3].

Humoral Theory

Hippocrates, a prominent figure in medicine during the period of 460-370 BCE, made noteworthy advancements in various aspects of medical knowledge, including understanding and treating epilepsy. The individual declined to accept supernatural justifications and advocated for a more logical and reasoned methodology. Hippocrates posited that epilepsy was attributable to a natural etiology, specifically localized inside the cerebral region. The individual in question created the notion of "humor," proposing that equilibrium in bodily fluids (including phlegm, blood, black bile, and yellow bile) may precipitate seizures. Although the humoral theory represented a shift from supernatural explanations, it lacked a neurological foundation [4].

The Contributions of Galen

Galen, a renowned Greek physician who lived from 129 to about 200 CE, further developed and elaborated on the concepts put forward by Hippocrates. The scholar made significant advancements in the humoral theory and postulated that epilepsy might be attributed to an inherent disruption in the equilibrium of the cerebral humors. The contributions of Galen had a profound and enduring impact on the field of medicine, shaping the prevailing medical beliefs for many centuries. One significant aspect of Galen's work was his support for the idea that epilepsy was caused by imbalances inside the body [9]. Nevertheless, these hypotheses failed to establish a definitive taxonomy for epilepsy, instead focusing on elucidating its underlying etiology.

The Renaissance and Enlightenment Periods

The Renaissance and Enlightenment epochs were characterized by a notable transition toward medical methodologies emphasizing empirical observation and anatomical investigation. Prominent medical practitioners such as Andreas Vesalius (1514-1564) and Thomas Willis (1621-1675) initiated investigations into the anatomical and physiological aspects of the brain. Willis, specifically, made noteworthy contributions to the comprehension of the neurobiological underpinnings of epilepsy. The contributions of his work established the fundamental basis for further inquiries into the illness [10]. In the 19th century, we have witnessed significant developments in American politics and semiotics. One notable phenomenon during this period was the Jacksonian March, which profoundly impacted the nation's political landscape. Additionally, semiotics emerged as a prominent academic discipline, contributing to the understanding of significant advancements in the classification of epilepsy that were observed during the 19th century. John Hughlings Jackson was a prominent British neurologist who significantly contributed to neuroscience. He is best known as Jackson (1835-1911), an eminent neurologist of English origin who conducted pioneering research in seizure phenomenology. The author proposed the notion of the "Jacksonian march," which elucidated the propagation of seizures throughout several cerebral areas, resulting in diverse motor and sensory manifestations. The emphasis placed by Jackson on the study of seizure semiology established the fundamental basis for comprehending various forms of seizures and their categorizations [7].

*The International League Against Epilepsy (ILAE) Emerged Throughout the 20th* *Century*

The foundation of the ILAE in 1909 marked a significant turning point in the classification of epilepsy during the 20th century. The ILAE has played a crucial role in standardizing the classification of epilepsy. The introduction of the concept of "epileptic syndromes" by the ILAE in 1960 involved the classification of epilepsies according to clinical symptoms, electroencephalography (EEG) findings, and causation. This development represented a notable deviation from prior classification systems, placing greater emphasis on a systematic and all-encompassing approach [6].

Contemporary Classification Systems

The impact of the ILAE remains significant in shaping the current classification of epilepsy. The latest taxonomy, implemented in 2017, enhances the comprehension of epilepsy by integrating progress in neuroimaging, genetics, and molecular biology. The significance of both seizure type and epilepsy syndrome classification is underscored, acknowledging the varied presentations of the illness [7]. The classification of epilepsy throughout history has undergone a significant transformation, progressing from supernatural explanations to a more empirical and scientifically grounded understanding. This statement highlights the persistent human endeavor to understand and classify this intricate neurological condition. Early classifications were initially based on superstitious beliefs and humoral ideas. However, the advancements made by influential personalities such as Hippocrates, Galen, Jackson, and the ILAE have played a significant role in developing modern classification systems. These contemporary systems now encompass a multidimensional approach.

Modern epilepsy classification systems

Within the domain of epilepsy, the categorization of this condition has undergone substantial development over time, resulting in a more refined and multifaceted framework. The categorization system created by the ILAE is widely regarded as one of the most prominent modern classification systems [8]. This section aims to conduct an in-depth examination of modern epilepsy classification systems, specifically focusing on the ILAE classification. It will highlight the fundamental ideas and criteria that serve as the foundation for these systems.

The Classification System Developed by the ILAE

The ILAE is a prominent worldwide association, including experts in the field of epilepsy. This organization has significantly influenced the development and structure of epilepsy categorization. The primary objective of this organization is to establish uniformity in the nomenclature and categorization systems of epilepsy. This improves our comprehension of the condition and promotes efficient communication among medical professionals, researchers, and those affected by epilepsy [9]. The ILAE implemented its latest categorization system in 2017, developed based on prior versions and incorporating the advancing understanding of epilepsy.

The Principles Underlying the ILAE Classification

The ILAE classification system is based on several fundamental concepts, all contributing to a thorough and nuanced comprehension of epilepsy. The ILAE classification system places significant emphasis on the clinical phenotype of epilepsy, considering the specific attributes of seizures and the accompanying symptoms [9]. Acknowledging the clinical manifestation is vital in customizing therapy and forecasting results. The identification of etiology, which refers to the fundamental cause of epilepsy, plays a pivotal role in the process of classification. The recognition is that epilepsy can manifest from diverse sources, including genetic mutations, anatomical abnormalities in the brain, or causes currently unknown (idiopathic) [10]. The precise identification of the underlying cause is crucial for determining the likely outcome and making informed choices regarding treatment. The classification system developed by the ILAE lays significant emphasis on the categorization of epileptic syndromes. The statement acknowledges identifiable patterns in how seizures are presented and the related characteristics, which remain consistent among individuals and can be categorized into distinct epileptic syndromes. The utilization of syndromic classification provides significant insights into the response to treatment and prognosis [10]. The utilization of EEG results has substantial importance in categorization, namely in the differentiation between focal and generalized seizures. The ILAE method incorporates the analysis of EEG characteristics as a crucial factor in the classification of epilepsy [11].

The ILAE Classification Criteria for Categorization

The ILAE classification system utilizes precise criteria and terminology to classify epilepsy. Several essential requirements are as follows: The ILAE categorizes seizures into two overarching classifications: focal onset seizures and generalized onset seizures. Seizures are categorized into subgroups according to their distinct semiology, encompassing motor symptoms, sensory symptoms, autonomic symptoms, and altered consciousness [12]. The classification of syndromes holds a fundamental position within the ILAE system [13]. Epilepsy syndromes are characterized by a distinct combination of clinical manifestations, age at which symptoms first appear, EEG results, and, frequently, genetic correlations. Several examples of epilepsy syndromes include childhood absence epilepsy, juvenile myoclonic epilepsy, and Lennox-Gastaut syndrome. The ILAE acknowledges a range of etiologies, encompassing structural, genetic, viral, metabolic, and immunological factors. Identifying the underlying etiology is essential to customize treatment approaches and get insight into the prognosis [14]. The classification scheme also considers the frequency of seizures, distinguishing between epilepsy characterized by frequent seizures (such as daily) and those characterized by uncommon or rare seizures. The ILAE classification system recognizes the heterogeneous nature of epilepsy in terms of its diverse outcomes and prognostic factors. Specific individuals experience prolonged periods of being seizure-free after treatment, but others may continue to have epilepsy that is resistant to treatment [15]. The classification considers the efficacy of antiepileptic drugs (AEDs) and surgical treatments in managing the condition. The text differentiates between those who achieve seizure remission with medication and those who experience drug-resistant epilepsy. The age of onset is a crucial factor to consider, as epilepsy can present itself at various points in an individual's life, ranging from early infancy to adulthood. There is a prevalence of some epileptic syndromes that exhibit a higher occurrence within distinct age cohorts [16]. The classification process is aided by identifying interictal discharges and distinct EEG patterns during seizures, as revealed by EEG data. The ILAE classification system is a comprehensive and adaptable foundation for comprehending epilepsy. Defining this complex condition is based on clinical phenotype, etiology, syndrome categorization, and EEG data, which collectively offer a systematic framework. Integrating these criteria into the ILAE classification serves multiple purposes, including facilitating precise diagnosis, informing treatment choices, and providing valuable prognostic and outcome-related information. The ongoing expansion of our understanding of epilepsy is accompanied by the continuous development of the ILAE categorization system, which is a flexible instrument that adapts to the progress made in research and therapeutic applications.

Advancements in neuroimaging

The utilization of neuroimaging techniques has been of great significance in the discipline of neuroscience, leading to a transformative impact on our comprehension of both the structural and functional aspects of the brain. Advanced technologies have facilitated the exploration of the complexities inherent in the human brain, leading to the acquisition of significant knowledge pertaining to diverse neurological conditions, such as epilepsy. The field of epilepsy has dramatically benefited from the ongoing advancements in neuroimaging techniques, including MRI and functional imaging modalities such as fMRI and PET. These techniques have played a crucial role in understanding and characterizing epilepsy, a prevalent and diverse neurological disorder characterized by recurring seizures. Utilizing these methods has not only enhanced our capacity to represent and determine the location of epileptic lesions visually. Still, it has also played a role in advancing more accurate and personalized categorization systems for epilepsy. This comprehensive study aims to examine the significance of neuroimaging techniques in the classification of epilepsy, with a focus on recent advancements and their substantial influence on enhancing our comprehension of epilepsy subtypes and approaches to therapy.

The Importance of Neuroimaging in the Classification of Epilepsy

The identification and determination of the specific location of lesions: The principal application of neuroimaging in epilepsy involves detecting and localizing structural irregularities within the brain that potentially contribute to seizures. MRI has been widely recognized as the preferred method for this objective. High-resolution structural MRI can detect and visualize minute abnormalities, such as mesial temporal sclerosis, cortical dysplasia, tumors, vascular malformations, and post-traumatic lesions. These pathologies have been identified as potential etiologies for the development of epilepsy. Accurate identification and precise location of these lesions are of utmost importance in directing surgical procedures in cases of drug-resistant epilepsy [17]. The differentiation of epilepsy types is a crucial aspect to consider, as epilepsy is not a singular condition but a collection of disorders exhibiting various causes and clinical manifestations. Neuroimaging plays a crucial role in differentiating between many kinds of epilepsy, including focal epilepsy and generalized epilepsy. Focal epilepsy, characterized by its origin in a distinct brain region, frequently exhibits observable structural irregularities on MRI scans, rendering these scans indispensable for classification [18]. In contrast, it should be noted that generalized epilepsy, which is defined by seizures that originate across the brain, may not exhibit any observable structural abnormalities. This highlights the significance of employing other neuroimaging techniques, such as functional imaging, in categorizing [16]. Functional imaging techniques, including fMRI and PET, have played a crucial role in elucidating the dynamic functional changes linked to epilepsy. These techniques offer valuable insights into the atypical brain networks and connection patterns implicated in seizure development and spread. The latest developments in resting-state fMRI have facilitated the identification of distinct resting-state networks linked to epilepsy. This has proven to be valuable in the categorization of epilepsy and the development of treatment strategies [19].

Neuroimaging has progressed beyond reliance on subjective visual examination with the introduction of quantitative imaging measurements. Quantitative imaging metrics, such as voxel-based morphometry and diffusion tensor imaging (DTI), accurately assess brain anatomy and connectivity. These measures have the potential to aid in the characterization of small changes in the brain, hence enhancing the accuracy of epilepsy subtype classification. As an illustration, DTI can detect deviations in the microstructure of white matter pathways, offering a valuable understanding of the fundamental pathophysiology [20]. Longitudinal neuroimaging investigations have yielded significant insights into the temporal dynamics of structural and functional alterations in individuals with epilepsy. By monitoring these alterations over time, medical practitioners and scholars can enhance the categorization of different subtypes of epilepsy and evaluate the efficacy of therapeutic interventions. Monitoring disease development and therapy response holds significant importance in refining classification methods and customization of treatments [21].

Latest Advancements in Neuroimaging Techniques to Classify Epilepsy

The field of neuroimaging data processing has experienced a notable incorporation of ML techniques in recent years, namely in ML and pattern recognition. ML methods, namely deep learning, can extract intricate patterns from neuroimaging data that may be invisible to human visual perception. The models mentioned above have demonstrated potential in automating epilepsy subtype categorization using structural and functional neuroimaging data [17,18]. Convolutional neural networks (CNNs) have been utilized in the detection of minor cortical anomalies in MRI scans, hence assisting in the categorization of focal epilepsy [22]. The integration of many neuroimaging modalities has emerged as a robust methodology in the categorization of epilepsy. The utilization of several imaging modalities, such as structural MRI, fMRI, PET, and other techniques, enables a thorough evaluation of the brain's structural and functional characteristics. Utilizing a multimodal method has promise in enhancing classification precision and facilitating a comprehensive comprehension of epilepsy [22]. The sensitivity and specificity of neuroimaging techniques have been improved through advancements in imaging sequences. For instance, high-resolution 7T MRI presents enhanced spatial resolution, which facilitates the identification of minor cortical anomalies that would have been overlooked when employing lower-field MRI systems [23]. Moreover, creating innovative contrast agents for PET imaging has enhanced the identification of molecular markers linked to epilepsy. This progress has paved the way for exploring fresh approaches in subtype categorization and formulating treatment strategies [24]. Connectomics, the scientific discipline dedicated to investigating the intricate networks of brain connectivity, has recently experienced a surge in prominence. The application of advanced network analysis approaches to functional and structural connectivity data has unveiled discernible network modifications in different subtypes of epilepsy. The results of this study hold the possibility of redefining the categorization of epilepsy by considering aberrations at the network level, offering a more thorough comprehension of the illness [23].

The primary objective of epilepsy categorization is to facilitate precision medicine, which involves customizing treatments for each individual based on their distinct subtype and underlying disease. Significant progress has been made in the field of neuroimaging, which has propelled us toward the realization of this objective. For instance, the discernment of distinct imaging biomarkers linked to the response to treatment enables doctors to make better-informed judgments on the selection and enhancement of therapy [24]. Neuroimaging techniques have been crucial in categorizing epilepsy, as it has yielded valuable information regarding the structural and functional irregularities linked to this intricate neurological condition. Neuroimaging has dramatically enhanced our comprehension of several subtypes of epilepsy, ranging from the accurate identification of lesion locations to the characterization of dynamic functional networks. In recent times, notable advancements have been made in various areas, including ML, multimodal imaging, sophisticated imaging sequences, connectomics, and the quest for precision medicine. These advancements have significantly enhanced our capacity to categorize epilepsy and customize treatment approaches for each patient. As we go, further investigation and advancement in the field of neuroimaging are expected to result in the development of increasingly advanced methodologies for classifying epilepsy. These developments will facilitate the progress of more precise and individualized classification systems and establish a foundation for more efficient treatment options, thereby enhancing the quality of life for those with epilepsy.

Genetic insights into epilepsy

Epilepsy is a complex and heterogeneous neurological disorder characterized by recurrent unprovoked seizures. Historically, epilepsy classification has been based primarily on clinical features and EEG findings. However, in recent years, the field of epilepsy research has undergone a profound transformation due to the growing recognition of the significant role genetics plays in the classification of epilepsy subtypes. Advances in genetic testing techniques, including next-generation sequencing (NGS), have unveiled many genetic mutations associated with various epilepsy syndromes. In this comprehensive review, we will elucidate the expanding role of genetics in epilepsy classification, emphasizing specific gene mutations linked to different epilepsy subtypes.

The Growing Role of Genetics in Epilepsy Classification

Precision diagnosis: The traditional approach to epilepsy classification, relying on clinical presentation and EEG patterns, often falls short in providing precise diagnoses, especially in cases with overlapping or atypical features. Genetics offers a more objective and accurate means of categorizing epilepsy subtypes by identifying the underlying genetic mutations responsible for seizure disorders. This approach allows for a finer-grained classification, aiding personalized treatment strategies [25].

Identification of novel epilepsy genes: The advent of NGS technologies, such as whole-exome sequencing (WES) and whole-genome sequencing (WGS), has enabled researchers to uncover novel epilepsy-associated genes at an unprecedented rate. These discoveries have expanded our understanding of the genetic basis of epilepsy and have the potential to redefine existing classification schemes [26].

Recognition of genetic epilepsy syndromes: Many epilepsy syndromes once considered idiopathic or cryptogenic have been linked to specific genetic mutations. For example, mutations in the SCN1A gene are associated with Dravet syndrome, while mutations in the KCNQ2 and KCNQ3 genes are linked to benign familial neonatal epilepsy (BFNE). These genetic insights have improved diagnostic accuracy and led to tailored treatments and prognosis predictions [27].

Specific Gene Mutations Associated With Epilepsy Subtypes

SCN1A (sodium voltage-gated channel alpha subunit 1): Dravet syndrome is a severe, intractable epilepsy syndrome that typically begins in the first year of life. Fever-induced seizures and developmental regression characterize it. Mutations in the SCN1A gene, which encodes the sodium channel Nav1.1, are the primary genetic cause of Dravet syndrome [27].

KCNQ2 and KCNQ3 (potassium voltage-gated channel subfamily q member 2 and 3): BFNE is a benign epilepsy syndrome that presents with neonatal seizures, often resolving within the first year of life. Mutations in the KCNQ2 and KCNQ3 genes that encode potassium channels have been identified as causative factors in BFNE [28].

CDKL5 (cyclin-dependent kinase-like 5): CDKL5 deficiency disorder is a rare epileptic encephalopathy characterized by early-onset seizures, severe developmental delay, and intellectual disability. Mutations in the CDKL5 gene, which is involved in neuronal development and synaptic function, are responsible for this disorder [28].

DEPDC5 (DEP domain containing 5): DEPDC5 mutations have been associated with familial focal epilepsy, often with cortical dysplasia. These mutations disrupt the mTOR (mammalian target of rapamycin) pathway, leading to abnormal cell proliferation and neuronal hyperexcitability. Understanding DEPDC5-related epilepsy has implications for targeted therapies [29].

GRIN2A (glutamate ionotropic receptor NMDA [N-methyl-D-aspartate] type subunit 2A): Mutations in the GRIN2A gene have been linked to various epilepsy syndromes, including autosomal dominant epilepsy with auditory features (ADEAF). Auditory and focal seizures characterize ADEAF. GRIN2A encodes a subunit of the NMDA receptor, which plays a crucial role in synaptic plasticity and neuronal excitability [30].

PNPO (pyridoxamine 5′-phosphate oxidase): Pyridoxamine 5'-phosphate oxidase deficiency is a rare metabolic disorder leading to seizures, developmental delay, and intellectual disability. Mutations in the PNPO gene disrupt vitamin B6 metabolism, essential for neurotransmitter synthesis [31].

SLC2A1 (solute carrier family two member 1): Glucose transporter type 1 deficiency syndrome (GLUT1-DS) is characterized by seizures, developmental delay, and movement disorders. Mutations in the SLC2A1 gene, which encodes the glucose transporter GLUT1, impair glucose transport to the brain, leading to neuronal dysfunction [32].

The role of genetics in epilepsy classification has grown significantly in recent years, with genetic testing technologies uncovering an array of mutations associated with various epilepsy subtypes. This transformation in our understanding of the genetic basis of epilepsy has several profound implications for clinical practice and research. First, genetics offers a more precise and objective means of classifying epilepsy subtypes, improving diagnostic accuracy, and facilitating personalized treatment strategies. Second, it has allowed for the recognition of genetic epilepsy syndromes, leading to tailored treatments and prognosis predictions. Third, ongoing genetic research continues to unveil novel epilepsy-associated genes, expanding our understanding of the underlying mechanisms of the disorder. As the field of genetics and epilepsy classification continues to evolve, clinicians and researchers need to stay updated on the latest genetic discoveries and their clinical implications. This knowledge not only enhances our ability to diagnose and treat epilepsy effectively but also paves the way for developing targeted therapies and precision medicine approaches to manage epilepsy.

Precision medicine in epilepsy

Epilepsy is a complex neurological disorder characterized by recurrent seizures, affecting over 50 million people worldwide. Traditionally, epilepsy treatment has followed a one-size-fits-all approach, with AEDs prescribed based on seizure type and EEG findings. However, epilepsy is a highly heterogeneous condition with diverse underlying causes and mechanisms. The advent of precision medicine has transformed epilepsy management by recognizing the importance of individualized treatment plans. This comprehensive review will explore the concept of precision medicine and its application in epilepsy management. Additionally, we will highlight how improved classification, based on genetics and other factors, contributes to personalized treatment approaches.

The Concept of Precision Medicine

Precision medicine, also known as personalized medicine, is an innovative approach to healthcare that recognizes each patient's unique genetic, molecular, and clinical characteristics. Instead of adopting a one-size-fits-all strategy, precision medicine aims to tailor medical treatment and interventions to the individual, optimizing therapeutic outcomes while minimizing adverse effects [33]. This approach acknowledges that individuals with the same disease may respond differently to treatment due to genetic, environmental, and lifestyle factors.

Application of precision medicine in epilepsy management: Precision medicine begins with a precise diagnosis and classification of the patient's epilepsy subtype. Advancements in genetic testing, neuroimaging, and EEG analysis have enhanced our ability to identify the underlying causes and mechanisms of epilepsy accurately [33]. Through genetic testing, specific gene mutations associated with epilepsy syndromes can be identified, leading to a more precise diagnosis. For example, SCN1A mutations are linked to Dravet syndrome [27], while mutations in KCNQ2 and KCNQ3 are associated with benign familial neonatal epilepsy [28]. Improved classification allows clinicians to select treatments that target the underlying cause or mechanism of epilepsy rather than relying solely on empirical AED trials.

Individualized treatment selection: Once a precise diagnosis is established, precision medicine enables the selection of AEDs based on the patient's specific epilepsy subtype and genetic profile. Some AEDs may be more effective or better tolerated for specific epilepsy syndromes or genotypes [34]. For instance, in patients with SCN1A mutations and Dravet syndrome, sodium channel-blocking AEDs like stiripentol have shown better efficacy [27]. In contrast, patients with KCNQ2 mutations may benefit from potassium channel openers like ezogabine [28].

Optimizing treatment response: Precision medicine involves continuous monitoring and adjustment of treatment plans based on individual patient responses. Biomarkers, including genetic markers and neuroimaging findings, can help assess treatment effectiveness and guide modifications [35]. For example, EEG monitoring can detect changes in seizure frequency and patterns, allowing for timely adjustments in medication dosages or the exploration of alternative treatment strategies. Precision medicine helps identify patients with drug-resistant epilepsy, in which seizures do not respond to standard AEDs. This knowledge is crucial for considering alternative treatment options, such as epilepsy surgery or neuromodulation therapies [33]. Genetic markers and clinical and neuroimaging data can aid in the early recognition of drug-resistant epilepsy, enabling the pursuit of more targeted interventions.

Development of novel therapies: Precision medicine research in epilepsy extends beyond existing treatments. It fosters the development of novel therapies, including gene therapies, targeted pharmacological agents, and neuromodulation techniques [32]. For example, the identification of specific genetic mutations associated with epilepsy has paved the way for gene therapies aimed at correcting these mutations [34]. This innovative approach holds promise for individuals with previously untreatable genetic epilepsies.

Improved Classification Contributes to Personalized Treatment

Tailoring AEDs to genetic profiles: Genetic information allows clinicians to select AEDs more likely to be effective for a specific patient's genetic subtype. For example, patients with SCN1A mutations might benefit from AEDs targeting sodium channels, such as fenfluramine [25]. Personalized AED selection minimizes the risk of adverse effects and maximizes therapeutic outcomes.

Disease-modifying therapies: In some epilepsy syndromes, disease-modifying therapies can target the underlying mechanisms rather than symptom control. Precision medicine facilitates the identification of suitable candidates for such therapies [35]. For example, individuals with genetic mutations leading to mTOR pathway activation may be candidates for mTOR inhibitors like everolimus [36].

Predicting treatment response: Improved classification and biomarker identification help predict how an individual will respond to treatment. This information enables early adjustments to treatment strategies, avoiding unnecessary medication trials and side effects [33]. Predictive biomarkers also inform the selection of alternative treatments for patients with drug-resistant epilepsy, such as responsive neurostimulation [37].

Enhancing clinical trials: Precision medicine enhances the design and success of clinical trials by ensuring that trial participants are more homogenous in terms of their epilepsy subtype. This improves the likelihood of detecting treatment efficacy and safety signals [34]. Targeted therapies developed through precision medicine are more likely to benefit patients with specific genetic or mechanistic subtypes. Precision medicine is revolutionizing epilepsy management by recognizing each patient's individual genetic, molecular, and clinical characteristics. Through improved diagnosis and classification, precision medicine enables the selection of personalized treatment strategies that target the underlying cause or mechanism of epilepsy. This approach optimizes treatment response, identifies drug-resistant epilepsy early, fosters the development of novel therapies, and enhances the success of clinical trials. As research in precision medicine continues to advance, healthcare providers and researchers need to stay updated on the latest discoveries in epilepsy genetics and biomarker identification. By embracing precision medicine principles, we can significantly improve the lives of individuals with epilepsy by providing tailored, effective, and safer treatment options.

AI in epilepsy classification

Epilepsy is a multifaceted neurological condition characterized by recurring seizures, impacting many individuals globally. Precisely identifying and categorizing epilepsy are paramount in informing therapeutic choices and enhancing patient prognoses. The amalgamation of AI and ML has significantly transformed epilepsy diagnosis and categorization. The utilization of AI-driven methodologies has promise in augmenting the precision and efficacy of these procedures, hence resulting in treatment solutions that are more tailored and impactful. This thorough analysis explores the integration of AI and ML techniques in diagnosing and categorizing epilepsy. Additionally, it will provide instances of developments in the field that AI has driven.

AI and ML Techniques in the Field of Epilepsy Diagnosis and Classification

The real-time detection and prediction of seizures is a prominent application of AI in epilepsy care. AI systems, namely deep learning models, possess the capability to evaluate EEG data to identify patterns associated with seizures [38]. CNNs have been utilized to accurately detect epileptic seizures in EEG recordings, as demonstrated by previous studies [39]. This technology can potentially enhance early intervention and patient safety. AI and ML systems have exhibited exceptional proficiency in classifying epilepsy subtypes using clinical data, neuroimaging, and genetic information. These algorithms have the potential to aid clinicians in achieving more accurate diagnoses. For instance, ML models have been trained to discern between focal and generalized epilepsy by utilizing EEG data, thereby assisting in determining appropriate treatment strategies. The utilization of AI in the automated analysis of EEG recordings is significantly transforming the process of interpreting EEG data. These tools can detect minor irregularities that could go unnoticed by human observers, resulting in more precise diagnoses. The utilization of AI-driven EEG analysis has the potential to aid in identifying the seizure onset zone, which is a crucial aspect of assessing individuals being considered for epilepsy surgery [37]. AI and ML methodologies are employed in creating predictive models that evaluate the likelihood of epilepsy-associated consequences, including the effectiveness of treatments, coexisting medical conditions, and overall well-being. These models facilitate healthcare professionals in making well-informed judgments and customizing treatment plans [40]. ML algorithms can estimate the probability of particular genetic variants that are linked to epilepsy, assisting in the process of genetic counseling and providing guidance for additional diagnostic examinations [39].

Illustrations of AI-Powered Progress in the Discipline

Deep learning models, specifically recurrent neural networks (RNNs) and CNNs, have demonstrated remarkable efficacy in automating the identification of epileptic seizures in EEG data. The models mentioned above can effectively assess extensive datasets and acquire intricate temporal patterns closely linked to seizures [38]. The efficacy of a CNN-based model in identifying seizures in extended EEG recordings was established by a research study, achieving a sensitivity rate of 94.7% and a specificity rate of 95.4%. AI algorithms are increasingly being utilized to evaluate structural MRI scans for the classification of focal epilepsy and the localization of epileptogenic areas. The algorithms above can detect minor irregularities that might be disregarded during eye examination [39]. ML algorithms have been devised to forecast the probability of particular genetic alterations linked to epilepsy by utilizing clinical and EEG data. These models can assist in prioritizing genetic testing and counseling initiatives [40]. AI's utilization in neuroimaging analysis plays a significant role in discovering and characterizing neuroimaging biomarkers, which can potentially enhance the diagnosis and categorization of epilepsy. These biomarkers may encompass assessments of brain connections, abnormalities in brain volume, and changes in brain function. There is ongoing development of predictive models utilizing AI and ML techniques to evaluate the response to treatment in individuals with epilepsy. These models can integrate a range of clinical and demographic variables to forecast outcomes and provide guidance for treatment choices. ML methodologies have been employed to forecast the response to particular AEDs, hence facilitating the selection of treatment options tailored to individual patients [38]. Incorporating AI and ML in diagnosing and categorizing epilepsy signifies a paradigm shift within the discipline. The technologies mentioned above can augment the precision and efficacy of seizure detection, enhance the classification of epilepsy subtypes, automate the processing of EEG data, construct predictive models, and uncover biomarkers derived from neuroimaging and genetics. Consequently, using AI-driven breakthroughs significantly enhances treatment techniques for persons diagnosed with epilepsy, leading to increased personalization and effectiveness. The adoption of AI and ML technologies is of utmost importance for doctors, researchers, and healthcare organizations, as they provide the potential to transform the field of epilepsy management significantly. The future of epilepsy care is anticipated to be marked by enhanced precision and individualization, resulting in improved patient outcomes and quality of life. This projection is based on the continuous advancements in AI algorithms and the availability of extensive datasets.

Challenges and controversies

Classification of epilepsy is a complex and evolving discipline crucial for guiding treatment decisions, prognosis, and research. While significant progress has been made in recent years, several ongoing debates and challenges persist, including issues related to the boundaries of specific syndromes and the need for standardization. This section will examine these challenges and controversies, followed by a discussion of possible future research and innovation directions.

Limits of Particular Epilepsy Syndromes

Defining the boundaries of particular epilepsy syndromes is one of the primary challenges of epilepsy classification. Clinical characteristics, seizure categories, and EEG patterns define epilepsy syndromes. However, epilepsy can manifest in various ways, making placing patients precisely into predefined categories difficult. For instance, some patients may manifest overlapping characteristics of multiple syndromes, making accurate classification difficult. This difficulty is especially evident when no identifiable genetic markers or EEG abnormalities are associated with a particular syndrome. When endeavoring to classify such patients, clinicians frequently encounter a gray area.

Genetic Complexity

Owing to genetic testing advances, genetics' significance in epilepsy classification has increased substantially. While this has provided valuable insights into the genetic underpinnings of epilepsy, it has also posed several challenging questions. Hundreds of genes are associated with various epilepsy syndromes, making epilepsy genetically diverse. When multiple genes are implicated in the same syndrome or novel genetic variants are discovered [25,26], it can be challenging to identify the genetic mutations responsible for an individual patient's condition. Specific genetic mutations can result in multiple epilepsy syndromes, thereby obscuring the distinctions between syndromes. For instance, mutations in the SCN1A locus can lead to Dravet syndrome, generalized epilepsy with febrile seizures plus (GEFS+), and other epilepsy phenotypes [27,28]. This genetic overlap makes classification more difficult.

Diagnostic Imaging Difficulties

Neuroimaging techniques, such as MRI, play a crucial role in the classification of epilepsy, especially in identifying structural abnormalities that may be the cause of seizures. Nonetheless, obstacles also exist in this field: Some structural lesions, such as small cortical dysplasias or modest hippocampal sclerosis, may be difficult to detect using a standard MRI. These subtle lesions can cause refractory epilepsy, and their identification is essential for accurate classification [23]. Radiologists and neurologists may interpret neuroimaging findings differently, resulting in inconsistencies in diagnosis and classification. Standardization of imaging protocols and enhanced training in neuroimaging techniques specific to epilepsy may help mitigate this challenge [24].

Standardization and Consistency

Classification of epilepsy is dependent on standardization. Consistency in diagnosing and classifying epilepsy is essential for accurate treatment and research. Nonetheless, standardization efforts encounter several obstacles: Despite the widespread acceptance of international classification systems such as the ILAE classification, variations in their implementation across regions and healthcare settings can contribute to inconsistencies in classification and treatment [5,6]. Clinical practice can vary between healthcare institutions and providers. Differences in diagnostic criteria, EEG interpretation, and imaging protocols can cause variations in classification [17].

Future directions

Despite these challenges and controversies, ongoing research and innovation offer promising directions for the future of epilepsy classification. Here are some insights into potential future developments: The future of epilepsy classification lies in embracing a precision medicine approach. This approach recognizes the individuality of each patient, considering genetic, neuroimaging, and clinical data to tailor treatment strategies. Advances in genetic testing and AI-driven analytics will play a crucial role in identifying precise epilepsy subtypes and guiding personalized treatments [38]. The development of advanced neuroimaging techniques, such as high-resolution MRI, PET, and magnetoencephalography (MEG), holds promise for improving the localization of epileptogenic zones and detecting subtle structural abnormalities [24]. Integrating AI algorithms for automated neuroimaging analysis will enhance the accuracy and consistency of structural and functional assessments. Ongoing genetic research will continue to uncover new epilepsy-related genes and mutations. This knowledge will contribute to improved classification and open doors to targeted gene therapies and disease-modifying treatments [26]. Genetic counseling and testing will become increasingly integral to epilepsy management. The utilization of big data and ML will become more prevalent in epilepsy classification. Large datasets encompassing clinical, genetic, EEG, and neuroimaging information will empower AI models to identify complex patterns and refine syndrome definitions [36]. These models may assist clinicians in making more accurate and consistent classifications. Efforts to standardize diagnostic criteria, imaging protocols, and EEG interpretation guidelines will continue to improve consistency in epilepsy classification [39]. International collaboration and the adoption of standardized practices will reduce regional variations in classification. A patient-centered approach to epilepsy classification will gain prominence. It involves actively involving patients in the classification process, considering their unique experiences, preferences, and treatment goals. Patient-reported outcomes and shared decision-making will become integral components of classification [39].

Areas of Research and Innovation to Watch for

To stay at the forefront of epilepsy classification, researchers and healthcare providers should closely monitor several ongoing research and innovation areas. Integrating AI and ML into diagnostic tools, such as EEG analysis software and neuroimaging platforms, will continue to evolve. These tools will aid in accurate classification and streamline the diagnostic process. Advancements in genomic medicine, including WGS and functional genomics, will shed light on the genetic underpinnings of epilepsy. Researchers should closely follow developments in genetic discoveries and their implications for classification. The search for reliable biomarkers in EEG and neuroimaging will remain a focal point. Innovations in imaging technologies, such as fMRI and MEG, will continue to improve the localization and characterization of epileptogenic zones. Establishing comprehensive epilepsy registries and increased data sharing among healthcare institutions will enable researchers to access diverse datasets for refining classification criteria and validating AI models [32]. Patient advocacy groups and initiatives focused on epilepsy awareness, and research will play a vital role in shaping the future of classification. Patients and caregivers are increasingly influential voices in driving research priorities and ensuring that classifications align with patient needs.

## Conclusions

In conclusion, this narrative review emphasizes the ongoing difficulties and debates surrounding the classification of epilepsy. It highlights the difficulty of delineating the boundaries of particular epilepsy syndromes, genetic heterogeneity, diagnostic imaging limitations, and the need for standardization. Despite these obstacles, the review highlights the significance of adapting to the ever-changing epilepsy classification landscape. Among the key conclusions is the increasing importance of genetics in classification, with genetic overlap and complexity necessitating ongoing research. Neuroimaging techniques of the future offer promise but standardization efforts are necessary. In addition, AI-driven innovations have the potential to result in more accurate and consistent categorization. Regarding the future, precision medicine approaches, sophisticated neuroimaging, genetic discoveries, and standardization initiatives will play crucial roles in refining the classification of epilepsy. Also essential are patient-centered care and advocacy. Adapting to these changes is essential for improving patient care, tailoring treatments, and advancing our comprehension of epilepsy, ensuring that individuals with this condition receive the most appropriate and effective care possible.
